# The anti-inflammatory effects of levocetirizine - are they clinically relevant or just an interesting additional effect?

**DOI:** 10.1186/1710-1492-5-14

**Published:** 2009-12-17

**Authors:** Garry M Walsh

**Affiliations:** 1 School of Medicine, University of Aberdeen, Aberdeen, UK

## Abstract

Levocetirizine, the R-enantiomer of cetirizine dihydrochloride has pharmacodynamically and pharmacokinetically favourable characteristics, including rapid onset of action, high bioavailability, high affinity for and occupancy of the H1-receptor, limited distribution, minimal hepatic metabolism together with minimal untoward effects. Several well conducted randomised clinical trials have demonstrated the effectiveness of levocetirizine for the treatment of allergic rhinitis and chronic idiopathic urticaria in adults and children. In addition to the treatment for the immediate short-term manifestations of allergic disease, there appears to be a growing trend for the use of levocetirizine as long-term therapy. In addition to its being a potent antihistamine, levocetirizine has several documented anti-inflammatory effects that are observed at clinically relevant concentrations that may enhance its therapeutic benefit. This review will consider the potential or otherwise of the reported anti-inflammatory effects of levocetirizine to enhance its effectiveness in the treatment of allergic disease.

## Introduction

The effects of histamine are exerted through three well defined classical G protein coupled histamine receptor subtypes termed H1R, H2R, and H3R [1] and the more recently described H4R [2]. Histamine signalling through H1R is responsible for the majority of the immediate manifestations of allergic disease. Levocetirizine (Xyzal^®^) is the single R-isomer of the racemic mixture piperazine H1R-antagonist cetirizine dihydrochloride in a once-daily 5mg formulation. The parent compound cetirizine (Zyrtec), a once-daily 10 mg formulation, is also an effective treatment for allergic disease being the most-widely used second-generation antihistamine worldwide. Levocetirizine is a selective, potent, oral histamine H1R antagonist that is licensed in Europe as tablets and oral solution for use in adults and children over 2 years of age for the symptomatic treatment of allergic rhinitis (including persistent allergic rhinitis) and chronic idiopathic urticaria. More recently, levocetirizine tablets under the trade name Xyzal have been approved by the Food and Drug Administration for use in adults and children over 6 years of age in the United States.

## Efficacy and safety

Levocetirizine is a potent antihistamine as demonstrated by its ability to inhibit cutaneous histamine-induced itching and the wheal and flare reaction [3-5]. The histamine-induced wheal and flare model in human skin is a widely-used reproducible and standardized methodology that gives an objective measure of the effectiveness of antihistamines in human subjects, together with any differences in onset and duration of action. The majority of these studies found levocetirizine to be the most potent of the antihistamines tested [5], including the parent compound cetirizine [6]. Large, well designed controlled clinical trials have demonstrated the efficacy of levocetirizine in adults with allergic rhinitis and chronic idiopathic urticaria [7,8], while well conducted studies have demonstrated levocetirizine to be safe and effective in young children with atopic rhinitis [9,10] or chronic urticaria [11]. Levocetirizine appears to have significant effects on nasal blockage [12,13]. The positive effects on nasal congestion are important findings as many antihistamines are ineffective in this regard. Indeed, histamine is not thought to be the primary cause of nasal congestion but a consequence of other mast cell-derived mediators including prostaglandin D2 and leukotrienes acting in concert [14]. The positive effect by levocetirizine on this important symptom of AR is likely due to its additional anti-inflammatory properties (see below).

In terms of its pharmacological profile levocetirizine exhibits rapid absorption and high bioavailability giving a fast onset and long duration of antihistaminic effect. These observed effects are mirrored by calculations of histamine H1 receptor occupancy that show a rapid and long-lasting presence of levocetirizine at its site of action. In terms of safety levocetirizine exhibits a low potential for drug interactions together with a lack of effect on cognition, psychomotor function and the cardiovascular system [15]. Indeed a recent study examined the sedative potential of a comprehensive battery of first, second and newer generation antihistamines (levocetirizine, desloratadine and levocetirizine) by calculating a proportional impairment ratio for each drug based on studies that used standardised objective methodology and psychometric tests. Levocetirizine had the lowest proportional impairment ratio of all the antihistamines reviewed, followed by fexofenadine and desloratadine respectively [16].

## Anti-inflammatory effects - In vitro

There is considerable interest in the effect of anti-inflammatory drugs on the pro-inflammatory processes responsible for the manifestations of allergic disease. Anti-inflammatory effects independent of H1-receptor blockade have been described for the majority of anti-histamines while the parent compound of levocetirizine, cetirizine has extensive well documented anti-inflammatory properties both in vivo and in vitro [17]. A number of recent in vitro studies have been conducted to assess whether levocetirizine has similar properties. Levocetirizine inhibited eotaxin-induced eosinophil transendothelial migration through monolayers of human dermal or lung microvascular endothelial cells in vitro at concentrations equal to or lower than those achieved in the clinical setting [18]. Physiologically-relevant concentrations of levocetirizine also inhibited both resting and GM-CSF-stimulated eosinophil adhesion to vascular cell adhesion molecule-1 (VCAM-1) under flow conditions in an in vitro model of the post-capillary venules [19]. Real time imaging revealed that the effect of levocetirizine on post-adhesion behaviour (detachment, flatness) contributed to its inhibitory action on eosinophil adhesion to rhVCAM-1. Other studies have also demonstrated in vitro anti-inflammatory effects by levocetirizine at therapeutically meaningful drug concentrations including inhibition of eotaxin production by endothelial cells [20] or inhibition of ICAM-1 and major histocompatability complex (MHC) class I expression by IFN-γ-stimulated keratinocytes together with modulation of histamine-dependent release of GM-CSF and chemokines by these cells [21]. Furthermore, histamine-induced, but not IL-4/TNFα-induced, VCAM-1 expression by nasal polyp-derived human fibroblasts was also inhibited by low concentrations of levocetirizine [22]. Levocetirizine does not appear to accelerate the rate of apoptosis-induction in eosinophils either in the presence or absence of viability-enhancing cytokines [18,23]. However, levocetirizine increased release of the metalloproteinase MMP-9, which is important in airway remodelling in asthma and the metalloproteinase inhibitors TIMP-4, and TIMP-1 together with a reduction in release of IL-7 and stem cell factor by lipopoylsaccharide-stimulated eosinophils. The former is important in T cell function and to some extent eosinophil function while stem cell factor is a key factor in mast cell proliferation. Another recent study demonstrated that both levocetirizine and cetirizine inhibited IL-8 and GM-CSF production by IL-1β-stimulated A549 epithelial cells. The latter is a cell line derived from type II malignant pneumocytes and positive inhibitory effects were only seen at rather high non-physiological concentrations of cetirizine or levocetirizine [24]. Physiologically-relevant concentrations of levocetirizine inhibited ICAM-1 expression and secretion of IL-6 and IL-8 in primary human nasal epithelial cells infected with human rhinovirus. Nasal epithelial cells treated with levocetirizine also exhibited significantly reduced rhinovirus titres and reduced NF-B activation [25].

These studies demonstrate in vitro anti-inflammatory effects by levocetirizine at low, physiologically-relevant concentrations on diverse cell types comparable to those reported for cetirizine. However, an obvious question is the extent to which these anti-inflammatory properties for a given antihistamine have any clinical impact in addition to that given by H1-receptor blockade. This question can only be answered by well conducted in vivo studies.

## Anti-inflammatory effects - in vivo studies

A number of reports do suggest that additional anti-inflammatory effects may be of relevance to the efficacy of levocetirizine. Ciprandi and colleagues [26] compared the effect of treatment with levocetirizine, desloratadine or placebo on changes in nasal inflammatory markers and nasal symptoms and airflow in seasonal allergic rhinitis patients during the pollen season. They demonstrated that levocetirizine, but not desloratadine or placebo, significantly decreased the number of eosinophils, neutrophils and IL-8 levels in nasal lavage samples during the pollen season while treatment with either antihistamine significantly reduced IL-4 levels. Furthermore, levocetirizine was also significantly more effective than desloratadine and placebo in attenuating nasal symptoms and in increasing nasal airflow from baseline in these patients. These findings suggest that levocetirizine-mediated improvements in nasal symptoms and airflow in patients with seasonal allergic rhinitis may be associated with attenuation of inflammatory markers in the nasal passages of these individuals. More recently levocetirizine and desloratadine were compared for their ability to inhibit allergen-induced wheal and flare in a double-blind, randomized, cross-over, placebo-controlled study in 18 allergic subjects [27]. This is an interesting approach as the use of allergen-challenge mimics the in vivo situation. Thus the elicited response involves mast cell degranulation and release of numerous vasoactive and pro-inflammatory mediators in addition to histamine. The authors evaluated the inhibitory activity of levocetirizine and desloratadine on the allergen-induced wheal and flare reaction at 1.5 h, 4 h, 7 h, 12 h and 24 h after administration at their respective therapeutic doses. Compared with placebo both antihistamines significantly inhibited allergen induced wheal and flare reactions. However, levocetirizine was more potent in its effect and also had a more rapid onset of action; most likely as a consequence of its higher receptor occupancy. The secretion of cytokines from lymphocytes, particularly Th2 cells, appears to be central to the establishment and maintenance of an allergic inflammatory response. It is of interest therefore that a recent study in patients with seasonal allergic rhinitis examined the effect of levocetirizine treatment on both symptoms and peripheral blood eosinophil numbers and lymphocyte subpopulation profiles. Compared with placebo levocetirizine treatment had significant positive effects on symptoms, reduced eosinophils and activated pro-inflammatory T cell numbers, namely: CD4+CD29+, CD4+CD212+, and CD4+CD54+. Interestingly, the authors also reported increased peripheral blood numbers of CD4+CD25+, a T cell subset that may include protective immunoregulatory (Treg) cells. The authors concluded that the in vivo changes in eosinophil and T cell subpopulations in the peripheral blood of seasonal allergic rhinitis patients treated with levocetirizine may contribute to improved clinical prognosis and also indicate important immunomodulatory effects for this drug [28].

In a recent study nasal challenge with adenosine 50-monophosphate AMP was shown to be a valid inflammatory marker of anti-allergic treatment efficacy in allergic rhinitis with a high degree of correlation with standardized nasal allergen challenge. AMP acts on mast cell adenosine (A2b) receptors, leading to cellular degranulation and release of pro-inflammatory mediators including histamine, cysteinyl leukotrienes, prostaglandins and IL-8. These authors further demonstrated in a randomized, double-blind, placebo-controlled, cross-over study that levocetirizine had significant effects on symptoms following nasal AMP challenge and also on the specific allergen challenge in patients with intermittent and persistent allergic rhinitis [29].

Evidence is accumulating that some second-generation antihistamines may benefit patients with allergic asthma as concomitant therapy [30]. An inhalation challenge with AMP induces bronchial hyper-responsiveness by acting indirectly via primed airway mast cells. This bronchial hyper-responsiveness correlates positively with eosinophilic asthmatic inflammation and atopic disease expression. One study found that single and short-term dosing of patients with atopic asthma with levocetirizine conferred improvements in bronchial hyper-responsiveness following AMP challenge, which was unrelated to pre-challenge airway calibre [31]. The authors concluded that further studies are indicated to evaluate the longer-term effects of levocetirizine on asthma exacerbations. A more recent randomized double-blind study reported positive effects by 5 mg levocetirizine given daily over eight weeks compared with placebo in patients with allergic asthma concomitant to allergic rhinitis with particular effects on quality of life parameters. Furthermore, use of rescue therapy (cromolyn and salbutamol) was significantly lower in the levocetirizine group. The authors concluded that their findings further support the theory of "united airway disease" [32] but emphasise that levocetirizine should not be considered a first-line medication for asthma but rather a useful add-on therapy in patients with allergic rhinitis with co-morbid asthma [33].

It is interesting to note that a study that demonstrated clinical improvement in chronic urticaria patients following levocetirizine treatment also reported a significant reduction in the levels of the circulating adhesion molecules P-selectin and E-selectin. The authors hypothesized that this observation may indicate a reduction in cell adhesion molecule expression by endothelial cells following levocetirizine treatment. This in turn might result in anti-inflammatory effects through inhibition of leukocyte adhesion and extravasation [34].

## Conclusions

There are now substantial numbers of well-conducted clinical trials that demonstrate that levocetirizine is an effective and well tolerated treatment for allergic disease in adults, children and infants. Studies investigating the mechanisms underlying the effects of H1-antihistamines have indicated that, in addition to their being a potent antihistamine, levocetirizine exhibits anti-allergic/anti-inflammatory effects, some of which may not be attributable to H1-receptor blockade. These anti-inflammatory activities are observed at clinically relevant concentrations, both in vitro and in vivo. Importantly a number of long-term studies (6-18 months) have reported long-term benefits by levocetirizine in adults and children not only in terms of positive symptom reduction but also on improvements in quality of life [10,11,15,33]. Although it is possible that these long-term effects of levocetirizine may also be a consequence of additional anti-inflammatory effects, this needs to be confirmed in future well-conducted studies. Moreover, the mechanism(s) by which the second and newer generation of antihistamines, including levocetirizine, block or inhibit the functions of key allergic response effector cells remains elusive.

## Competing interests

The author has received research funding, honoraria, travel support and expenses from the manufacturers of levocetirizine: UCB Pharma SA, Belgium.
